# Using E-GARCH to Analyze the Impact of Investor Sentiment on Stock Returns Near Stock Market Crashes

**DOI:** 10.3389/fpsyg.2021.664849

**Published:** 2021-07-27

**Authors:** Sze Ting Chen, Kai Yin Allison Haga

**Affiliations:** ^1^China-ASEAN International College, Dhurakij Pundit University, Bangkok, Thailand; ^2^International Master Program in Asia-Pacific Affairs, College of Social Sciences, National Sun Yat-sen University, Kaohsiung, Taiwan

**Keywords:** investor sentiment index, E-GARCH, small-cap stock effect, yield, financial assets

## Abstract

**Purpose:** Investor sentiment, the willingness of market participants to invest, is a difficult concept to measure. Exploring the relationship between investor sentiment and stock returns can reveal how investor sentiment affects the operation of the stock market. Such an understanding can assist market participants in making more rational investment decisions based on market laws. Such an understanding can also assist regulators in their roles of supervision and policy making.

**Methodology:** Although the E-GARCH model has the advantage of considering volatility clustering, it has not previously been used to investigate the impact of investor sentiment changes on the Shanghai Composite Index's market return. This research therefore applies the E-GARCH approach to data from 2015 to 2018, to explore the influence of investor sentiment on the return rate of the Shanghai Composite Index.

**Main Findings:** There are three main findings. First, when the investor sentiment is increased by the same amount, the rate of return before a stock market crash will have a smaller increase than the rate of change after the crash, which is a new finding. Second, the rate of return on stocks is susceptible to emotional sentiment, rather than simply depending on stock price. Third, the tendency of retail investors to follow the crowd is less in periods of pessimism than it is in periods of optimism, which, in turn, can push up stock yields.

**Application:** Based on these research results, this article can provide insights to understand how investors' subjective judgments on future earnings affect their investment behavior and how great the impact is on the market. At the same time, it can help investors make more rational investment decisions based on an understanding of market laws, and help regulators with guidance for their supervision and policy making.

**Originality/Value:** This paper contributes to the theory of the investor sentiment index, improving the index construction method by adding two sentiment proxy indicators: investor activity ACT and stock market leverage level. After constructing the sentiment index and comparing it with the stock market index (Shanghai Composite Index), the fit is found to be improved.

## Introduction

In July 2020, the “China Securities Journal” pointed out that China's A-share market “is necessary and conditional to enter a healthy bull market,” which would be necessary for promoting economic recovery, attracting foreign investment and competing with other countries. Beijing wants to replicate the V-shaped rebound seen in the US stock market, in the hopes that the resulting wealth effect and emotional boost will be effective in solving many domestic economic challenges during the COVID-19 crisis. The current bull market in China is driven by liquidity/policies, with capital flowing from bonds and bank deposits into stocks. Without strong corporate earnings support, market sentiment would tend to be fragile. Moreover, several factors are also potential risks for severe market volatility: disappointing economic data in the second half of the year, corporate earnings in the second quarter and guidance for the second half of the year, geopolitical risks and potential sanctions brought about by Sino-US trade war negotiations and/or political turmoil in Hong Kong, worsening floods in China, and a second wave virus outbreaks (Feng and Liu, 2020). Unlike the 2015 A-share bull market (driven by leverage and margin financing), local investors have thus-far continued to store funds in the market, given that China's domestic bond yields and deposit rates have fallen.

The coronavirus epidemic impacts the real economy. Gong et al. (2020) noted the poor 2020 quarterly reports of some listed companies. Because of the impact of the epidemic on the A-share market, the short-term market has fluctuated sharply before stabilizing. How long this effect will last depends on the evolution of epidemic. From a fundamental point of view, despite the severity of the epidemic, its impact on the real economy is limited, with many stocks having “panic” declines that may reverse in time. Regarding the current valuation level of the A-share market, Chen et al. (2019) argued, even prior to the outbreak of the epidemic, that the current valuation of most individual stocks had reached a historic bottom. The current investor structure in the A-share market is very unbalanced, however, with retail investors accounting for over 90% and institutions accounting for under 10%. Whereas, institutional investment centers on the future growth expectations of listed companies, retail investment tends to be more speculative and influenced by emotion. This large amount of retail investment may amplify swings in the A-share market, with investors first being overly optimistic based hearsay of good performance, and then being panicked by market drops, with a reluctance to reinvest until there is a recovery.

China A shares peaked in the bull market of early 2016 and have yet to regain those heights (Hu and Wang, 2018; Yang, 2018). The sharp rise and fall in 2015 was caused by the Central Bank of China lowering the reserve requirement ratio and the interest rate in April 2014, for the first time in over 2 years. With interest rates dropping, the A-share market rose sharply. Measuring from June 2014, the market would double in less than a year. Such intense speculation created, in turn, fears for the A-share market's outlook. The A-share market began to plummet in mid-June 2015, with multiple crash days for the stock market. The government intervened when the Shanghai Composite Index fell to 4,000 points, but this did not produce a recovery. The market was still far from its bottom, and it was not uncommon to see 1,000 shares fall to their government-imposed daily limit. By 2018, A-shares showed a downward trend throughout the year, owing to factors such as an economic downturn, market volatility, and continued geopolitical uncertainty (e.g., the Brexit process, Sino-US trade disputes and other factors). The Shanghai Composite Index fell 24.59% and the Shenzhen Component Index fell 34.42%, both nearing records with the second largest drops in their histories. In early 2019, the downward trend reversed, but the recovery had stalled by May.

The Chinese capital market is 40 years old (beginning in 1981), but remains immature when compared with the capital markets of developed countries. Whereas, most participants in developed markets are institutional investors (Clark and Monk, 2017), about 70% of Chinese market participants are individual investors, with only 30% being institutional investors. Irrational speculative trading behavior is also more widespread in China's A-share market, with a tendency for investors to chase short-term ups and downs. Also, the information available to the public is very limited, which makes the A-share market more susceptible to rumors. All these factors, as well as others, can easily cause serious deviations in the perception of the market. Therefore, it is particularly important to study the relationship between the stock market and investor sentiment. Past literature has shown that investor sentiment affects stock prices (Renault, 2017; Qadan and Nama, 2018). In subsequent studies, researchers also proposed that investor sentiment affects the value of corporate bonds and options (Bethke et al., 2017; Seok et al., 2019). This research further improves the method of constructing the investor sentiment index by adding two sentiment proxy indicators (investor activity ACT and stock market leverage level) to look at how the investor sentiment fluctuation index is related to stock yield before and after a stock market crash.

Traditional financial theories view investors as generally rational, and consider that rational arbitrageurs can counteract the influence of irrational investors on prices (Daniel and Titman, 1999; Kozak et al., 2018). The core theories of traditional finance include the modern asset portfolio theory of Markowitz (1952), the popular MM theorem proposed by Franco and Merton (1958), the capital asset pricing theory (CAPM) proposed by Sharpe (1964), the efficient market hypothesis proposed by Fama (1970), the option pricing theory proposed by Black and Scholes (1973), and the arbitrage pricing theory (APT) proposed by Ross (2013), etc. Since 1950, traditional finance theories gradually developed into a discipline with a strict logic and unified analytical framework. Since the 1980s, however, financial markets exhibited many anomalies that could not be explained by existing financial theories, such as a momentum effect, an under-reaction anomaly, a small-cap stock over-reaction anomaly, and closed fund discounts behavior.

As a result, behavioral finance theory began to replace traditional finance theories, as it better explains these financial anomalies. Behavioral finance theory employs cognitive psychology and treats investors as being rationally limited in terms of cognition, emotion, and attitude (Day, 2016; Mushinada, 2020). Investors therefore respond wrongly to market noise and cause systematic cognitive biases. Behavioral finance theory considers how these irrational noise traders affect the operation of efficient markets (De Long et al., 1990; Cuong et al., 2019). Investor sentiment (De Long et al., 1990; Kumari and Mahakud, 2016) is one of the irrational factors that causes noise trading. Studying the relationship between investor sentiment and market returns will therefore contribute to behavioral finance theory. This study can also help investors make more rational investment decisions based on a better understanding of market rules, while also helping regulators in guiding their supervision and policy making.

### Research Gap

In recent decades, an increasing number of scholars have studied the impact of investor sentiment on financial asset prices (Zheng, 1999; Kelly and Ahmad, 2018), as well as on investor sentiment and stock prices (Baker and Wurgler, 2006; Smales, 2017). But most of these research works have examined developed countries. Is the situation in the emerging economies consistent with the results for developed countries? Moreover, few scholars have studied China's Shanghai Stock Exchange Index (SHCOMP) before and after stock market crashes. In addition, the SHCOMP is more representative than China's CCTV Watch Index, because the CCTV Watch Index is calculated through a survey of only 60 securities companies and consulting companies in Mainland China. With the continuous development of the capital market, the influence of these 60 institutions in the market will continue to weaken, so it is doubtful whether they can be accurately representative of overall market sentiment. Moreover, In the past, the literature has only talked about the relationship between investor sentiment and stock prices, but it rarely mentioned about whether the relationship between the two before and after the stock market crash was different from usual.

### Research Results

This paper not only improves the index construction method by adding two sentiment proxy indicators: investor activity ACT and stock market leverage level but also find that the coefficient before the stock market crash was smaller than that after the stock market crash, in comparing an increase in investor sentiment vs. an equally-sized decrease, the sentiment increase had less effect on the market rate of return before a stock market crash than the decrease had after a crash.

## Literature Review and Hypotheses

### Research Background

Neoclassical economic theory assumes that people are essentially rational, that information is openly disclosed, and that the market is effectively operated. In other words, people can use available information to rationally invest and consume, and the securities market can respond quickly and accurately to new information. Also, securities prices fully reflect all market information, with market competition making security prices shift from to a new balance in response to independent and random new information.

In the real world, however, the hypothesis of neoclassical economics theory is incomplete. Securities prices cannot fully reflect all the information in the market. New market information may not immediately impact the securities market, nor may the investors' response to that information be rational. Neoclassical economics ignores important aspects of human behavior and psychology. For example, psychological changes affect human decision-making, including investment decisions and consumption behavior. The earliest research on investor sentiment was based on human psychological factors (Watson, 1912). After that, behavioral finance became a field of study that evolved into modern investor sentiment theory. The prospect theory of Kahneman and Tversky (1979) focuses on investor behavior from a psychological perspective, assuming that retail investors are more susceptible to emotional factors or cognitive biases, which lead to unreasonable trading behaviors.

There is no standardized definition of investor sentiment. According to Baker and Wurgler (2006), investor sentiment is defined as “a belief about future cash flows and investment risks that is not justified by the facts at hand.” Similarly, Stein (1996) also defined sentiment as a systematic deviation of investors' expectations of the future. Investor sentiment seeks to measure market participants' willingness to invest or expectations for their investments. Although traders experience the undeniable influence of investor sentiment, measuring it presents the challenge of attempting to quantify a feeling (i.e., a sentiment). Moreover, different individual investors may not have the same sentiments. Despite the measurement challenges, it is well-recognized that investor sentiment is a necessary component of stock market behavior, with real explanatory power. In economic activities, sentiment is an uncertain factor, which affects investors' subjective judgments on future returns. This in turn affects its investment behavior and thus has a great impact on the market.

### Investor Sentiment and Financial Assets

Early research claimed that noise traders were not important to the formation of financial asset prices (Fama, 1965; Stephen, 1976; Khrennikova, 2016). Talwar et al. (2021) looked at the decision-making process of investors from the perspective of psychology and pointed out that individual investors could easily be affected by emotional factors or cognitive biases, leading to unreasonable trading behaviors. Baker and Wurgler (2006) also proved that investor sentiment could affect stock returns. Behavioral finance theory sees two forces as determining financial asset prices: noise traders and rational arbitrageurs. De Long et al. (1990), Gruber (2011) noted that traders chasing noise may be overactive or inactive, so their behavior could induce stock prices to deviate from their fundamentals. Irrational investors can over-react good news, but they can also ignore under-reported bad news (Brown and Cliff, 2004; Hirota and Sunder, 2016). Shleifer and Vishny (1997) proposed the theory of arbitrage limitation, which argues that the levels of irrational investment behavior and market noise trading are too high to be fully offset by arbitrageurs. As a result, rational arbitrageurs may reduce their positions or withdraw from the market, thereby further weakening the stabilizing effect of arbitrage and causing asset prices to deviate even further from their intrinsic value. Hence, from the perspective of behavioral finance, investor behavior can be explained by psychological perspectives and bullish/bearish sentiment indexes (Ryu et al., 2017).

Investor sentiment refers to the investment intentions and expectations of investors. Chau et al. (2016) identify such sentiments as the cause behind excessive fluctuations in risk tolerance that are too pessimistic or too optimistic in relation to asset forecasts. Whether pessimistic or optimistic, sentiment can cause an asset's price to deviate from its intrinsic value. The effect is hard to quantify, however, and questions (such as how high this sentiment is or how rapidly it is currently changing) are complicated by the wide differences in interpretations between investors, due to factors ranging from investment style to wealth status. De Long et al. (1990) and Han et al. (2016) proposed a noise trading model, due to the difficulty of predicting the mood of noise traders. They viewed noise trader sentiment as being always present within in the financial market, posing a systemic risk factor that could begin to noticeably affect asset pricing at any time.

Other researchers have tried to understand investor sentiment by combining the ideas of psychology and finance. Ye et al. (2020) noted that sentiment generates when investors rely on their own imperfect understanding of the market and their own cognitive psychology. Brown and Cliff (2004) and Haritha and Uchil (2019) consider investor sentiment as representing the investors' personal expectations for the market's rate of return. For example, investors with high optimism have higher expectations of yield than pessimistic investors have. Baker and Wurgler (2006) and Piccoli and Chaudhury (2018) have linked investor sentiment with a psychological speculative tendency. Rising investor sentiment raises interest in speculative trading, regardless of the differing arbitrage capabilities of the stocks in question. Such speculation therefore has a cross-sectional effect upon the entire market.

To summarize, we agree that modern investors have access to so much market information media that it does influence whether they form bullish or bearish expectations of the stock market's trend in the future. This current research on investor sentiment adopts quantitative methods, using several proxy indicators to approximate investor sentiment. A review of the literature reveals that these indicators fall into two categories: direct indicators of investor sentiment which are also known as an intuitive sentiment indicators, and indirect indicators which are also called objective sentiment indicators.

#### Investor Sentiment Indicator: Investors Intelligence Index, Bullish Sentiment Index, TURN

Six indicators are primarily used by scholars to measure the intuitive sentiment of investors, particularly in China. (1) The Investor Intelligence Index, or the II Index, has been updated weekly since 1964 and is compiled by Chartcraft Company. Lee et al. (2002) and Jitmaneeroj (2017) have used this index to study investor sentiment. (2) The Friendship Index is compiled by HARDADY. The statistical samples of the index are all institutional investors, including funds, insurance, private equities, and mainstream media. (3) The Haodan Index has been published since 1997 and is the first investor sentiment survey index published in mainland China. (4) The CCTV Watch Index has both daily and weekly frequency data. (5) The Bullish Sentiment Index (BSI) was designed by Cui (2013). (6) The Sina Long Short Index (SinaISI) is compiled by Sina Finance.

Seven proxy indicators of investor sentiment are primarily used by scholars. (1) The closed-end fund discount rate (CEFD) is the most common proxy variable to be used by Western scholars. Neal and Wheatley (1998) regressed the yields of large-cap and small-cap stocks on the New York Stock Exchange (NYSE) from 1933 to 1998 with the discount rate of closed funds over the same period. They found that the discount of closed-end fund could predict the differences between the yields of different types of stocks. When Brown and Cliff (2005) regressed the return rates of large-cap stocks and small-cap stocks over shorter periods from 6 months to 3 years, however, they found that the discount rate of closed funds could not significantly predict the return rate of stocks. (2) The turnover rate (TURN) refers to the frequency of stocks changing hands, as found in the market transaction data. Whereas, trading volume is an absolute quantitative indicator, the turnover rate is a relative quantitative indicator (Lee et al., 2009; Cohen et al., 2016). Baker and Stein (2004) used this indicator to research investor sentiment. They found that when the turnover rate (volume) in the market increases, it is often because the market is more dominated by irrational investors, causing a short-term overvaluation of the market, which indicates that the future rate of return may be low. (3) The Advance-Decline Line Index (ADL) is defined as the ratio of rising stocks (ADV) to declining stocks (DEC) per day, as found in the market performance index. Brown and Cliff (2005) have used this indicator. (4) The financing interest rate belongs to the market leverage index. Financing is the transaction of borrowing funds to buy securities. (5) The securities lending interest rate, which also belong to the market leverage index, is the transaction behavior of borrowing and selling securities. Scholars have used a combination of the financing rate indicator and the securities rate indicator to research investor sentiment (Brown and Cliff, 2004; Chen et al., 2016). (6) The number of new investors is an indicator of investor activity, and represents, to some extent, the views of potential investors who have not yet entered the market (over-the-counter investors). (7) The Investor Sentiment Index is a synthetic index constructed by Zhang et al. (2018). It combines the market turnover rate and closed-end fund discount rate as an objective sentiment indicator.

Nevertheless, when Kim et al. (2014) conducted an empirical study on the Sentiment Index compiled by Investors Intelligence Communications, they concluded that investor sentiment was useless, with no significant impact on market yields in either the short or long term. So more complex models were needed. Brown and Cliff (2005) and Zhang et al. (2018) established a VAR model for investor sentiment and market yield. They found that investor sentiment does have some predictive power for the long-term stock returns, although they agreed with Sot & Statman that this was not the case for short term yields. Baker and Wurgler (2006) and Bekiros et al. (2016) further found that investor sentiment on a cross-section can predict a variety of stock returns. They pointed out that when investor sentiment was higher (lower), the yields of small-cap stocks, performance-loss stocks, and high volatility stocks in the market were lower (higher), while large-cap stocks, performance-profitable stocks, and low-volatility stocks enjoyed a higher (lower) the rate of return. Engle (2001) studied the dynamic impact of institutional investors on stock returns by constructing a Markov-Switching-GARCH model, and found that an increase in the positions of institutional investors could change the volatile structure of market returns in the short term. In other words, the contribution of institutional investors to market stability was verified.

### Investor Sentiment and Stock Yield

Studies have shown that emotions affect stock returns, with investor optimism and pessimism leading to stock price fluctuations (Baker and Wurgler, 2006; Li et al., 2020). Stambaugh et al. (2012) and Renault (2017) found that, in the months following a high-mood period, the long-term anomaly strategy was more profitable than following a low-mood period. Sun et al. (2016) found that, during a recession, investor sentiment had little ability to predict stock returns. Investor sentiment can also change the perception of investors, affect their investment decisions, and lead to changes in stock prices (Stambaugh et al., 2012; Sun et al., 2016).

Cheng and Liu (2005) chose a preference index that represents investor sentiment and then used a VAR model to analyze investor sentiment and stock market returns. The results show that the medium-term investor sentiment index has more influence on the volatility of stock market returns than short-term investor sentiment has. The short-term sentiment index is much more impacted by market fluctuations in the return rate.

An alternative to the VAR model used by Cheng and others is the T-GARCH model used by Fang (2010) and An et al. (2018). The results show that when the market performs poorly, the higher (in a negative sense) is the sentiment index of retail investors, large investors, and institutional investors, and the lower is the daily yield of the Shanghai Stock Exchange. When the market performs well, the better is the investor sentiment and the higher is the daily yield of the Shanghai Composite Index. The T-GARCH model shows that investor sentiment has a good positive predictive ability for stock returns.

Chi et al. (2012) used the Extended Kalman Filter Method to establish their investor sentiment index. They found that investor sentiment has significantly more impact on small-cap stocks than on large-cap stocks. As for the impact of yield on investor sentiment, the yield of large-cap stocks will have a greater impact. Their other finding is that the fluctuations in investor sentiment are more predictive of market returns than the sentiment index itself.

To summarize, after investors receive market information, they will form a bullish or bearish expectation for the market trend. During periods of rising sentiment, investors' expectations for the market outlook will become more optimistic, stimulating high speculative trading and increasing market volume. This will, to a certain extent, promote the overall rise of the whole stock market index. During periods of downward periods of investor sentiment, investors' are more pessimistic about the market outlook, so demand for speculative transactions is weak, which can cause the stock index to drop further. Based on this, we propose our first research hypothesis:

*H*_1_: The more changed is the investor sentiment, the higher the stock market yield

The Leverage Effect commonly discussed in the field of finance produces an asymmetry between positive and negative shocks in the general financial time series. When a company's debt remains unchanged, a lower stock price for the company corresponds to lower shareholder equity. Therefore, the greater the company debt/shareholders' equity, the greater the company's residual risk arising from uncertainty about future cash flow. This effect from a negative shock is greater than for a positive shock. Accordingly, we propose our second research hypothesis:

*H*_2_: The negative impact of investor sentiment on the market yield is greater than the positive impact.

In addition, when studying the correlation between the investor sentiment index and stock returns, some issues have to be further considered. The overall situation of the stock market is always changing, and the influencing factors upon stock return are very complicated. In fact, no small set of factors is able to fully explain the market behavior. In the face of such a volatile market, is the impact of investor sentiment index on stock yields constant? Considering the radical surge and collapse of China's A-shares in 2015, within the sample period studied in this paper, we can also refine and analyze the influence of investor sentiment changes on stock returns before and after that crash. We thus propose our third research hypothesis:

*H*_3_: When increasing investor sentiment is of the same magnitude, the range of increase of the market yield before the stock market crash is smaller than that after the stock market crash.

## Methodology

### Sample and Data Collection

The sample data used in this article come from the official website of China Securities Depository and Clearing Corporation Limited (CSDC) and the Wind Financial Terminal. The time span is from January 1, 2015 to December 31, 2018, using all weekly data, for a total of 1,040 sets of data. This time period is selected because we consider that on January 1, 2015, the broad market was continuing a long bear market trend since 2009, where various indexes had downward trends. It was not until April 2015, when the central bank initiated its first RRR cut in over 2 years, that the broad market index began to stabilize. Two more rate cuts occurred in June and November of that year. Then in 2016, the market experienced a sharp spike and falling slide. Finally, by 2018, the market remained relatively stable with no bull or bear trends across the year, although the market had experienced continuous fusing at the beginning of the year. Therefore, by looking at the 4-year market as a whole, the market had experienced a relatively complete bull-bear cycle, which improves the reliability of the analysis and conclusions drawn in this article.

### The Selection Process of Emotional Agent Indicators

First, we built an investor sentiment index. Constructing an effective investor sentiment index requires selecting a true proxy index for investor sentiment. Following the standard practice in the literature, we first screened the primary indicators. This screening takes into consideration the possible lead-lag effect of proxy indicators. Baker and Wurgler (2006) indicate that this lead-lag effect of proxy variables of investor sentiment means that these variables can reflect investor sentiment in different periods. We analyze the correlation between the market index and the above-mentioned preliminarily screened sentiment proxy variables and their respective first-order lag variables. Then, we take the variable with the highest correlation coefficient as the corresponding final emotion proxy variable.

To carry out a follow-up empirical study of investor sentiment and stock market returns, this study uses the investor sentiment synthetic index SENTIMENT (Baker and Wurgler, 2006) as the independent variable. The explained variable is the return rate of the stock market. The raw data of the market is selected from the Shanghai Stock Exchange Composite Index (SHCOMP) and the current yield of the Shanghai Stock Exchange (Fang, 2010). This article selects only objective sentiment indicators to represent investors' sentiment in the market, and does not use intuitive indicators.

Intuitive sentiment indicators are generally obtained from specific questions in interviews or questionnaires; the various indexes are then constructed to measure investor sentiment based on the statistical results of investor answers. Although intuitive sentiment indicators can directly measure the feelings and expectations of investors, the validity of such data is still questioned by many industry experts and scholars. There are two main reasons. First, investor ideas may not necessarily be implemented in practice. When investors are interviewed, the views they may express about the future are subject to change. The noise in the market will affect investor decisions, and they may not end up investing in accordance with the opinions expressed in their interviews. Second, it is doubtful whether the sample of an interview survey is representative. For example, the CCTV Watch Market Index is calculated through a survey of 60 securities companies and consulting companies in Mainland China. However, with the continuous development of the capital market, the influence of these 60 institutions in the market will continue to lessen and make them unrepresentative of market sentiment. Based on the above two reasons, we believe that what investors ultimately “do” reflects market sentiment better than what they “think” they will do. In fact, various objective transaction data in the financial market contain the indicators of the sentiment of investors. Hence, this article only uses these objective sentiment indicators.

The construction of our comprehensive indicator of investor sentiment index (SENTIMENT) is as follows. The primary sentiment indicators we have used are market performance indicators (ARMS), market trading indicators (turnover rate, TURN), market activity indicators (investor weekly activity, ACT), and market leverage indicators (financing balance/free float market value ratio of the financing subject, LEVERAGE). The above-mentioned preliminary selected emotional proxy variables and their respective first-order lag variables and market index, were then analyzed for individual correlations. As Baker and Wurgler (2006) noted, these various proxy variables of investor sentiment may each have a different lead-lag effect; we therefore took the above-mentioned preliminary selected emotional proxy variables and their respective first-order lag variables and the market index, and then analyzed them individually for correlations. The variable with the highest correlation coefficient was then taken as the corresponding final emotion proxy variable. Based on these selections, we then use principal component analysis to construct the investor sentiment index of this article.

There are many objective sentiment indicators that characterize investor sentiment. We refer to Fang (2010) to divide the indicators that reflect objective emotions into 4 sub-categories: Market performance indicators (ARMS), Market transaction indicators (turnover rate, TURN), Market Activity Indexes (Investor Weekly Activity, ACT), and Market leverage indicators (the ratio of financing balance to the free market value of the financing subject, LEVERAGE).

#### Market Performance Indicators: ARMS

In addition to paying attention to the rise and fall (range) of the index after the close of each day, most of the stock investors will take note of how many stocks rose and how many fell each day, in order to perceive the strength and weakness of the stock market performance. One of the most common indicators is the vacancy index (amount of change ratio, ADL), which measures the number of stocks rising and falling each week, with the following ratio formula:

(1) $$\begin{array}{l} ADL_{t}=\frac{ADV_{t}}{DEC_{t}}\; \end{array}$$

This ratio measures the number of stocks whose closing prices had risen (*ADV*_*t*_) or fallen (*DEC*_*t*_) in week *t*. When the market sentiment is strong, investor enthusiasm is relatively high and the stock market often shows a general upswing. This upswing means that the *ADL* ratio will be large. When the market sentiment is down, it will suppress investor enthusiasm to a certain extent. A growing number of declining stocks will lower the *ADL* ratio. However, the simple measure of the number of rising and falling stocks does not take into account the trading volume of those corresponding stocks (VOLUME). To this end, we make a slight modification to the expression of the Advance-Decline Line (ADL) Index, choosing ARMS as the index to measure market performance, and defining it as:

(2) $$\begin{array}{l} ARMS_{t}=\frac{ADV_{t}/{VOLUME\text{\_}ADV}_{t}\;}{DEC_{t}/{VOLUME\text{\_}DEC}_{t}}\; \end{array}$$

Here, *VOLUME_ADV*_*t*_ represents the transaction amount of rising stocks and *VOLUME_DEC*_*t*_ represents the transaction amount of falling stock prices, in week *t*.

#### Market Transaction Indicators: Turnover Rate (TURN)

Turnover rate refers to the frequency with which stocks are bought and sold in the market during a unit of time, so it reflects the liquidity of stocks. When investor sentiment is high, transactional demand leads, driving a race toward stocks that appear likely to generate a quick profit. Therefore, the more active the stock is, the higher the turnover rate should be. In contrast, when investors are depressed, their trading reduces greatly. Their trading behavior will also tend to be conservative, and market transactions will be less, resulting in a low turnover rate. Therefore, the turnover rate index of the stock market can be used to reflect the strength of trading demand and can also be used as an indicator for measuring investor sentiment. We defines the turnover rate as:

(3) $$\begin{array}{l} TURN_{t}=\frac{\text{Number of traded }(\text{Shanghai A }{\text{Shares}}_{\text{t}}+\text{Shenzhen A }{\text{Shares}}_{\text{t}})}{\text{Free floating market value of }(\text{Shanghai A }{\text{Shares}}_{\text{t}}+\text{Shenzhen A }{\text{Shares}}_{\text{t}})} \end{array}$$

#### Market Activity Index: Investor Weekly Activity (ACT)

Regarding the selection of indicators for investor activity, Zhang and Yang (2009) selected the number of newly opened accounts in A-shares. This article uses the Investor Weekly Activity (ACT) indicator. There are two main reasons for this decision. First, measuring A-shares is difficult to changes made by the China Securities Regulatory Commission (CSRC) during our study's test period. The CSRC revised its “Securities Account Business Guide,” in April 2015, to allow investors to open A-share accounts at different 20 different brokerage firms; it then revised the policy again the following year, limiting then number to 3. With our sample data covering this time period, these policy changes will result in a distortion of the new investor account opening data. The second reason for not using the A-shares indicator is the problem of unavailable data. At present, the CRSC provides no direct announcement of the investor's weekly activity index. The data that the CSRC does publish (with weekly frequency) are: the number of investors at the end of the period, the number of position holders at the end of the period, and the number of investors who participated in the transaction during the period. Among these three provided data, the number of investors at the end of the period is similar to the number of investors holding positions at the end of the period. We choose to focus on the simple number of investors, because short positions are also a manifestation of attitude. Thus, we define the Investor Weekly Activity (ACT) based on the existing published data:

(4) $$\begin{array}{l} ACT_{t}=\frac{\text{Number of investors participating in transactions during Perio}{\text{d}}_{\text{t}}}{\text{Number of investors at the end of Perio}{\text{d}}_{\text{t}}}\;. \end{array}$$

#### Market Leverage Indicator: Financing Balance/Free Market Value of Financing Subject (Leverage)

In more-developed securities markets, the volume of margin trading and securities lending accounts for a large proportion of the total market transactions. These volumes are thus reflective of how leveraged the market is. Before 2010, China's A-share market only allowed unilateral long positioning. Since 2010, A-shares both long and short positions can be held parallel. A-share margin trading has undergone four large expansions. There are now 948 underlying securities, accounting for about 30% of the total number of A-shares in Shanghai and Shenzhen. However, the current securities lending business is still limited to selected securities firms. Since these firms only have funds in localities and a small amount of stocks, there remain fundamental problems and the growth of this business is slow. Over the same period, only 31 stocks out of 948 double financing target/subjects were securities financing subjects. Among the nearly 940 billion yuan of double financing, securities lending was only 3.5 billion yuan (<0.5%) With this number being essentially negligible, we only consider the impact of the financing balance and define the stock market leverage level (LEVERAGE) as:

$$\begin{array}{l} \text{LEVERAG}{\text{E}}_{\text{t}}=\frac{\text{Financing balanc}{\text{e}}_{\text{t}}}{{\text{Free floating market value of financing subject}}_{\text{t}}}\;. \end{array}$$

### Analytical Procedures

In order to analyze and predict volatile and asymmetric yield fluctuations, this article uses GARCH (1,1) (Bollerslev, 1986) and E-GARCH (Nelson, 1991). These are the two most widely used asymmetric univariate models of conditional volatility for investigating the impact of investor sentiment changes on stock yield. The asymmetric effects on conditional volatility of positive and negative shocks of equal magnitude can be captured in different ways by the exponential GARCH or EGARCH (McAleer and Hafner, 2014). Of GARCH, EGARCH, PGARCH & TGARCH, each model has a divergent purpose with normal error distribution techniques to measure the volatility of investor sentiment, specifically, by using GARCH, ERARCH. Moreover, one of the main advantages of E-GARCH is the model's logarithm of volatility (Mohsin et al., 2019; Salamat et al., 2020). Therefore, during the estimation period, no parameter limitation is required. When estimating the simplest univariate GARCH (1,1), the estimating program usually limits alpha and beta to greater than zero. This is ideal, but in the EGARCH model, such restrictions are not required.

The change of the investor sentiment index is taken as the explanatory variable in the empirical analysis, and the yield sequence of the Shanghai Composite Index is taken as the explanatory variable. Before modeling the time series and regression analysis, it is generally necessary to perform a stationarity test to avoid a spurious regression. So we conducted a stationarity test to ensure that the time series of the variables in the model are stable. The financial time series data do not have a stable mean, but do tend to be relatively stable in stages that are followed by drastic fluctuations. Simple linear models therefore also need to be tested by ARCH. The ARCH model is a statistical model for time series data. The model describes the variance of the current error term or innovation term as a function of the actual size of the error term in the previous time period.

After the test, we found that the residual sequence does not meet the independence requirement of OLS, so the value estimated by OLS is not unbiased. Therefore, this paper uses GARCH family of models to correct the residuals. The GARCH model requires that all of the coefficients be positive, and that the degree of positive and negative shocks must be consistent. After careful consideration, we selected the E-GARCH model. This model was proposed by Daniel (1991) to solve the problem of asymmetric impacts of positive and negative shocks on market returns in the field of financial analysis. Since it also solves the strict non-negative constraint influence of the GARCH model on parameters, E-GARCH is more suitable for our study. The specific results are shown in Table 1.

**Table 1:** The correlation coefficients of the market index SHCOMP and eight primary sentiment indicators.

**LEVERAGE**	**ACT**	**TURN**	**ARMS**	**LagLEVERAGE**	**LagACT**	**LagTURN**	**LagARMS**
0.771	0.899	0.811	0.302	0.775	0.891	0.815	0.301
^***^	^***^	^***^	^***^	^***^	^***^	^***^	^***^

*Source: This research collated*.

The “***” means significance at the 1% level. It is clear that the market index has a significant relationship with all eight of the above variables, with each correlation coefficient being far above 0. In comparing each variable with its lagged version, two of the four benefit from lagged analysis. The four sentiment indicators selected for this article are therefore LagLEVERAGE, ACT, LagTURN and ARMS. We next use principal component analysis to weigh the four variables and construct the investor sentiment index (SENTIMENT). We implement this process through SAS programming, to obtain the results shown in the following Table 2:

**Table 2:** Eigenvalues of the correlation matrix and its cumulative contribution to variance.

	**Eigenvalues**	**Difference**	**Proportion**	**Accumulation**
1	2.7537	1.8452	0.6885	0.6884
2	0.9086	0.6261	0.2271	0.9156
3	0.2826		0.0706	0.9862

*Source: This research collated*.

According to the results in Table 3, we can write the factor expressions of the first and second principal components (1),

(5) $$\begin{array}{l} Prin_{1}=0.532LagLEVERAGE+0.582ACT+0.571LagTURN\\ \;+0.228ARMS\; \end{array}$$

(6) $$\begin{array}{l} Prin_{2}=-0.716LagLEVERAGE-0.075ACT-0.146LagTURN\\ \;+0.970ARMS\; \end{array}$$

Then, according to the weights of the cumulative contributions of the first two principal components, we weighted the corresponding coefficients in the expressions of *Prin*_1_ and *Prin*_2_ and obtained the weighted average as the coefficient of the final emotional composite index to arrive at the final sentiment comprehensive index (SENTIMENT). The specific operation rules are as follows.

**Table 3:** Factor loading of the first three principal components.

	**Prin1**	**Prin2**	**Prin3**
LagLEVERAGE	0.533	−0.176	0.826
ACT	0.582	−0.075	−0.332
LagTURN	0.571	−0.146	−0.454
ARMS	0.238	0.970	0.056

*Source: This research collated*.

If we take the first T principal components, the expression of the principal component is *Prin*_*i*_ = ∑λ_*ki*_*X*_*i*_, (*k* ≤ T). It is also known that the variance contribution degree of the *k*_th_ principal component is $\sigma_{k}^{2}$. So the expression of the final emotional synthesis index is:

(7) $$\begin{array}{l} \text{Prin}=\frac{\sum_{k=1}^{T}\lambda_{k1}\sigma_{k}^{2}}{\sum_{i=1}^{T}\sigma_{i}^{2}}\;X_{1}+\frac{\sum_{k=1}^{T}\lambda_{k2}\sigma_{k}^{2}}{\sum_{i=1}^{T}\sigma_{i}^{2}}\;X_{2}+\ldots \\ \;+\frac{\sum_{k=1}^{T}\lambda_{kn}\sigma_{k}^{2}}{\sum_{i=1}^{T}\sigma_{i}^{2}}\;X_{n} \end{array}$$

Referring to (2), we finally get the expression of the investor sentiment index SENTIMENT used in this article:

(8) $$\begin{array}{l} {SENTIMENT}_{t}=0.333{LagLEVERAGE_{t}}_{-1}+0.42ACT_{t}\\ \;+0.39{LagTURN}_{t-1}+0.436ARMS_{t}. \end{array}$$

### Descriptive Statistics

We select the value of investor sentiment innovation (DSENTIMENT) as the explanatory variable of the empirical analysis model, which is define over a period *t* as:

(9) $$\begin{array}{l} {\text{DSENTIMENT}}_{\text{t}}={\text{SENTIMENT}}_{\text{t}}-{\text{SENTIMENT}}_{\text{t}-1} \end{array}$$

If *DSENTIMENT*_*t*_ > 0, then this means that the newly generated investor sentiment over period *t* is positive (i.e., new optimism). Similarly a *DSENTIMENT*_*t*_ < 0 means that the newly generated investor sentiment is negative (i.e., new pessimism).

The raw data of the explanatory variable is based on the Shanghai Composite Index (SHCOMP), with the current return rate of the Shanghai Composite Index (RetSHCOMP) as the explanatory variable. Also, we use the logarithmic rate of return to express the current rate of return. So the market rate of return in period *t* is expressed as:

(10) $$\begin{array}{l} {\text{RetSHCOMP}}_{t}=ln{\text{SHCOMP}}_{t}-{ln\text{SHCOMP}}_{t-1} \end{array}$$

The results of descriptive statistical analysis of the market return data are summarized in Table 4.

**Table 4:** Basic descriptive statistics of return rate data.

	***N***	**Mean**	**Standard deviation**	**Sum**	**Minimum**	**Maximum**
RetSHCOMP	205	0.0015	0.0349	0.3097	−0.1429	0.0909

### Stationarity Test

In this section, the time series of the explained variables is {*RetSHCOMP*_*t*_}. The time series of the explanatory variables is {*DSENTIMENT*_*t*_}. Therefore, we first perform unit root tests on these two sequences. This article uses the ADF inspection method. In the ADF test, the null hypothesis and alternative hypothesis are respectively defined as:

$$\begin{array}{l} H_{0}:\rho -1=0\;H_{a}:\;\rho -1<\;0 \end{array}$$

Here, the null hypothesis *H*_0_ means that the original time series is not stationary. Then in performing the tests of the time series, {Z}, we conduct the following regression:

(11) $$\begin{array}{l} Z_{t}-Z_{t-1}=\left(\rho -1\right)Z_{t-1}+\beta_{0}+\beta_{1}t+\beta_{2}\Delta Z_{t-1}\\ \;+\beta_{3}\Delta Z_{t-2}+v_{t} \end{array}$$

We implement the ADF stationarity test through SAS. The results of the test for the time series of the explanatory variable *RetSHCOMP*_*t*_ and of the explanatory variable *DSENTIMENT*_*t*_ are shown in Tables 5, 6.

**Table 5:** Unit Root Test Results of RetSHCOMP.

**Categories**	**Lag**	**Rho**	**Pr < Rho**	**Tau**	**Pr < Tau**	***F***	**Pr > *F***
Zero Mean	0	−175.04	0.0001	−12.36	<0.0001		
	1	−162.41	0.0001	−8.98	<0.0001		
	2	−160.30	0.0001	−7.51	<0.0001		
Single Mean	0	−175.39	0.0001	−12.35	<0.0001	76.25	0.0001
	1	−163.14	0.0001	−8.98	<0.0001	40.28	0.0001
	2	−161.75	0.0001	−7.53	<0.0001	28.25	0.0001
Trend	0	−175.40	0.0001	−12.32	<0.0001	75.88	0.0001
	1	−163.15	0.0001	−8.95	<0.0001	40.08	0.0001
	2	−161.78	0.0001	−7.50	<0.0001	28.13	0.0001

**Table 6:** Unit Root Test Results of DSENTIMENT.

**Categories**	**Lag**	**Rho**	**Pr < Rho**	**Tau**	**Pr < Tau**	***F***	**Pr > *F***
Zero Mean	0	−199.33	0.0001	−14.09	<0.0001		
	1	−157.72	0.0001	−8.86	<0.0001		
	2	−164.97	0.0001	−7.60	<0.0001		
Single Mean	0	−199.33	0.0001	−14.05	<0.0001	98.71	0.0001
	1	−157.73	0.0001	−8.84	<0.0001	39.10	0.0001
	2	−164.98	0.0001	−7.58	<0.0001	28.75	0.0001
Trend	0	−199.62	0.0001	−14.03	<0.0001	98.39	0.0001
	1	−158.64	0.0001	−8.85	<0.0001	39.14	0.0001
	2	−166.38	0.0001	−7.58	<0.0001	28.76	0.0001

Tables 5, 6 show that the time series for the two variables under study in the first question reject the null hypothesis of “H_0_: the original series has a unit root (that is, the series is not stationary).” The time series {*RetSHCOMP*_*t*_} and {*DSENTIMENT*_*t*_} are therefore stationary series.

### E-GARCH Model Testing

After confirming that these two time series are both stationary, the next step is to establish a linear regression model to examine the impact of investor sentiment changes {*DSENTIMENT*_*t*_} upon the current return rate of the market {*RetSHCOMP*_*t*_}. To do so, we first perform the Ljung-Box-Q test on the return sequence {*RetSHCOMP*_*t*_}. The test results are shown in Table 7.

**Table 7:** {*RetSHCOMP*_*t*_} serial white noise autocorrelation test results.

**Lag**	***Q* statistics**	**Degree of freedom**	**Pr > *Q***	**Autocorrelation coefficient**					
6	5.20	6	0.518	0.140	0.052	0.013	0.026	0.031	−0.027
12	13.67	12	0.323	−0.001	0.004	0.096	−0.065	−0.149	0.057
18	15.07	18	0.657	−0.050	−0.035	0.000	0.048	0.016	0.006
24	23.55	24	0.488	0.081	0.029	0.168	−0.004	−0.004	−0.034

We can see that none of the Q statistic values can reject the null hypothesis of a white noise sequence. The market return sequence {*RetSHCOMP*_*t*_} is therefore approximately a white noise sequence with no linear autocorrelation. Therefore, we first construct a simple OLS linear model [hereafter referred to as “model (7)”]:

(12) $$\begin{array}{l} \text{RetSHCOMP}=\text{c}+\beta_{1}\text{DSENTIMEN}{\text{T}}_{\text{t}}+\varepsilon_{\text{t}} \end{array}$$

The OLS linear regression model requires that the residual sequence {ε_*t*_} be independent and uniformly distributed. Unfortunately, Financial Time Series usually do not have a stable mean. While most time series are relatively stable in stages, they are often accompanied by severe fluctuations. Because there is an ARCH effect in the stock returns of every bank, the OLS with HAC estimation is not completely correct (Newey and West, 1987). We therefore need to run an ARCH-effect test (Lagranger Multiplier test) on model (7). The judgment index used is the LM statistic. The LM test runs the following regression:

(13) $$\begin{array}{l} \varepsilon_{t}^{2}=\beta_{0}+\beta_{1}\varepsilon_{t-1}^{2}+\beta_{2}\varepsilon_{t-2}^{2}+\ldots +\beta_{m}\varepsilon_{2}^{t-m}+e_{t} \end{array}$$

The original hypothesis is *H*_0_, which means that there is no ARCH effect in the residual sequence { ε_*t*_ } up to the order *m* (that is, β_1_ = β_2_ = … = β_*m*_ = 0) and the alternative hypothesis is *H*_*a*_(that not all of the β are equal to 0). The LM test results of model (7) are shown in Table 8 below.

**Table 8:** Model (7) ARCH test of residuals.

**Order**	***Q***	**Pr > *Q***	**LM**	**Pr > LM**	**Order**	***Q***	**Pr > *Q***	**LM**	**Pr > LM**
1	8.58	0.0034	8.52	0.0035	7	103.22	<0.0001	60.75	<0.0001
2	42.37	<0.0001	36.58	<0.0001	8	115.30	<0.0001	60.74	<0.0001
3	48.92	<0.0001	37.16	<0.0001	9	126.20	<0.0001	62.90	<0.0001
4	69.28	<0.0001	41.49	<0.0001	10	130.49	<0.0001	66.38	<0.0001
5	69.27	<0.0001	46.02	<0.0001	11	133.37	<0.0001	66.55	<0.0001
6	95.21	<0.0001	56.22	<0.0001	12	134.22	<0.0001	66.93	<0.0001

It can be seen that the *p*-values corresponding to LM statistics up to a 12th order lag are all <1%, which strongly rejects the null hypothesis *H*_0_. This means that the residual sequence {ε_*t*_} does have a high-order ARCH effect. Therefore, it does not meet the independence requirement of the OLS linear regression equation for the residual sequence. We also know that, under the ARCH effect, the β value estimated by OLS linear regression is biased and also ineffective. In order to obtain an unbiased estimate of β, this paper selects the GARCH (1,1) family model to correct the residuals and improve the model (7), in agreement with the procedure of Mohsin et al. (2020) which considers the GARCH (1,1) model to be the best fit model for obtaining volatility.

Assuming that the time series {ε_*t*_} follows the standard GARCH(m,s) model for the time series {ε_*t*_}, we obtain the following expression:

(14) $$\begin{array}{l} \{\begin{array}{c} \text{Mean equation}:\varepsilon_{t}=\sqrt{h_{t}}v_{t},\left\{v_{t}\right\}\;Zero\;mean\;white\;noise\;sequence\\ \text{ Variance equation}:h_{t}=a_{0}+\sum_{i=1}^{m}a_{i}\varepsilon_{t-1}^{2}+\sum_{j=1}^{s}\beta_{j}h_{t-j} \end{array} \end{array}$$

With the strict constraints of model (9):

(15) $$\begin{array}{l} s.t.\{\begin{array}{c} \begin{array}{c} a_{0}>0,a_{i}\geq 0,\beta_{j}\geq 0\\ \; \end{array}\;\\ \sum_{i=1}^{\max\left(m,s\right)}a_{i}+\beta_{j}<1 \end{array} \end{array}$$

Our research finds that the main constraint of the GARCH model is its higher requirements for the coefficients in the model. Another pair of problems is that the response must be positive and the negative financial shocks must be symmetrical. In fact, in a typical financial time series, negative shocks tend to be stronger than positive shocks. This asymmetry is called the leverage effect in finance. Regarding the issue of the impact of asymmetric shocks, scholars have appropriately extended the traditional standard GARCH model. The more popular extended GARCH models include the T-GARCH model and the E-GARCH model. The E-GARCH model was proposed by Daniel (1996). It not only solves the problem of the asymmetry of positive and negative shocks on market returns, but also solves the constraints that the parameters of the GARCH model be non-negative. Furthermore, The EGARCH model is popular, among other reasons, because it can capture both asymmetry, namely the different effects on conditional volatility of positive and negative effects of equal magnitude, and leverage, which is the negative correlation between returns shocks and subsequent shocks to volatility (McAleer and Hafner, 2014, p. 96.) Mohsin et al. (2020) considered how EGARCH (1,1) assesses the impact of leverage (negative or positive shock) on the unpredictability of bank stock returns. In addition, EGARCH (1,1) uses a logarithmic model of conditional variance, because logarithmic values can be positive or negative, thus avoiding the GARCH model's restriction against non-negative coefficients. We therefore use E-GARCH (1,1). The EGARCH model expression of the current return rate of the market (RetSHCOMP) and investor sentiment changes (DSENTIMENT) are written as:

(16) $$\begin{array}{l} \{\begin{array}{c} \text{Mean equation}:RetSHCOMP_{t}=c+\beta_{1}DSENTIMENT_{t}+\varepsilon_{t}\\ \text{where }\varepsilon_{t}=\sqrt{h_{t}}v_{t},\left\{v_{t}\right\}\;Zero\;mean\;white\;noise\;sequence\\ \text{ Variance equation }:\ln(h_{t})=a_{0}+a_{1}\left|\frac{\varepsilon_{t-1}}{\sqrt{h_{t-1}}}\right|+\phi {\;}_{1}\ln\left(h_{t-1}\right)+\theta {\;}_{1}\;\frac{\varepsilon_{t-1}}{\sqrt{h_{t-1}}}\; \end{array}\; \end{array}$$

Here, if the asymmetric effect coefficient θ_1_ is significantly below 0, it indicates that the impact of the shock is a leverage effect. In addition, the impact of the shock is asymmetric if θ_1_ ≠ 0. Also, there are no non-negative constraints on other parameters in the model.

## Results

After choosing the E-GARCH (1,1) model as the modification scheme of model (7), and the final regression results areas Tables 9, 10:

**Table 9:** Parameter estimation results of model (11) (1).

**Variable**	**Estimate**	**Standard Error**	***t* value**	**Approximate Pr > |t|**
C	0.0026	0.0018	1.44	0.1492
Q	0.0252	0.0063	4.07	<0.0001
*a*_0_	−0.1231	0.0791	−1.56	0.1109
*a*_1_	0.1418	0.0754	1.88	0.0601
ϕ_1_	0.9824	0.0112	88.32	<0.0001
**θ**_**1**_	−1.1901	0.7014	−1.70	0.0897

**Table 10:** Parameter estimation results of model (11) (2).

**SSE**	**Log likelihood**	**AIC**	**Total R party**	**Normality test**	**Pr > Chi-square**
0.2360	441.4553	−870.9104	0.0436	1.8229	0.4020

The above results produce three observations. First, the EGARCH model did not reject the null hypothesis that the residual sequence is white noise, which supports that the model estimation is well-realized. Second, in the mean value equation, the β coefficient is significantly above 0 throughout the sample time, which shows that there is a positive relationship between the market return (RetSHCOMP) and changes in investor sentiment (DSENTIMENT). Therefore, when investor sentiment generates new optimism (pessimism), the market rate of return tends to correspondingly increase (decrease). This proves that our hypothesis H_1_ does not reject the null hypothesis, and is supported. Third, it can also be seen from the parameter estimation results of Model 11 that the asymmetric effect coefficient θ_1_ is significantly below 0. This shows that, over the entire time period from 2015 to 2018, the impact of negative sentiment on the market return is greater than the impact of positive sentiment, indicating that there is a leverage effect in the market. As in Kasman's et al. (2011) research, EGARCH assessed the impact of leverage (positive or negative shock) on the unpredictability of bank stock returns. This proves that hypothesis *H*_2_ is supported, with the negative impact of investor sentiment on the market return being greater than the positive impact.

### Sectional Inspection Results Before and After the Stock Market Crash

The input data from 2015 contain a sharp spike and crash in China's A-share market. In order to analyze the data in better detail, we divide the sample data (from 2015 to 2018) into two time periods: before and after the stock market crash on June 12, 2015. Then, we build an E-GARCH (1,1) model of the market return sequence {*RetSHCOMP*_*t*_} on the investor sentiment change sequence {*DSENTIMENT*_*t*_} based on the (1,1) model for the above two time periods. The final parameter estimation results from the model are shown in Table 11.

**Table 11:** Parameter estimation results of the model (11) before and after the stock market crash.

	**Before the stock market crash**			**After the stock market crash**		
**Variable**	**Estimate**	***t*** **-value**	**Approximate Pr>** |**t**|	**Estimate**	***t*** **-value**	**Approximate Pr>** |**t**|
C	0.0032	1.33	0.1845	−0.0023	−0.63	0.5273
□	0.0191	2.57	0.0102	0.0478	3.61	0.0003
*a*_0_	−0.2362	−1.29	0.1973	−1.7703	−6.1	<0.0001
*a*_1_	0.0042	0.04	0.9693	0.4221	1.4	0.1619
ϕ_1_	0.9671	40.37	<0.0001	0.7415	16.76	<0.0001
θ_1_	−1.4972	−1.81	0.0783	−1.0444	−1.05	0.2932

From the results shown in Table 11, the β coefficient is significantly >0, both before and after the stock market crash. This shows that the market return rate had a positive correlation with the changes in investor sentiment. When there is a positive (negative) investor sentiment with new interest, the market return rate tends to increase (decrease). This result is the same as the estimated result of the whole stage. Also, we found that the β coefficient before the stock market crash was significantly smaller than that after the crash. This means that, when investor sentiment is increased (reduced) by the same magnitude, the rate of increase (decrease) in the market rate of return before the stock market crash is smaller than the rate of change in the market rate of return after the stock crash. This proves that hypothesis *H*_3_ is supported.

## Conclusion and Discussion

In China's stock market, fluctuations in the investor sentiment index have a profound impact on stock market yields. To a certain extent, this means that the effectiveness of the Chinese securities market is insufficient, with many other factors affecting the operation of the market. The reasons for this may include the imperfect system of China's securities market (Xie, 2016; Zhanga and Yaob, 2016), the need for stronger supervision of regulatory authorities, and the professionalism level of the investment participants. China's capital market has developed rapidly in recent years, expanding its market value, transaction volume, and company listings. If compared with the securities markets of developed countries in the West, however, the problems and deficiencies in China's securities markets are clear. Be it from the perspective of laws and regulations, of market systems, or of market efficiency, it is precisely because of these shortcomings that investor sentiment has such a significant impact on yields in China's securities market.

Studying various factors that affect stock returns, predicting and measuring returns of various types of financial products, and exploring the relationship between risk and return – all these have become important topics in finance. Concerning modern investment portfolio theory, classic financial theories lack explanatory power for many real-world stock price fluctuations. The academic community should strengthen its analysis of the many practical problems in the financial market, from the perspective of behavioral finance theory in emerging economies, as well as strengthening the completeness of capital asset theory and systematic development (Cassetti et al., 2020). This would help promote the development of the entire financial discipline. Focusing on the impact of investor sentiment on the yield of assets can guide improvements to the market and to the investment philosophy of investors. Policymakers, investors, and researchers are most interested in understanding stock returns and market performance, and how it interacts with return predictors. The Chinese government wants more foreign capital flow and wants to understand its impact on the market (Howes et al., 2017). As an emerging economy with unstable policies, with a fast changing environment, and with markets that have not yet been fully developed, there is a wide range of explanatory variables that may affect Chinese stock returns.

In addition to combining the investor sentiment index research of many prior scholars, this article further constructs a new investor sentiment index that adds two sentiment proxy indicators: investor activity ACT and stock market leverage level LEVERAGE. The final constructed sentiment index is found to produce a better fit than the broader market index (Shanghai Composite Index). We established the above-mentioned E-GARCH(1,1) model on the return sequence and on investor sentiment index changes to conduct an empirical analysis of this issue. Our study found that investor sentiment, whether optimistic or pessimistic, has a significant impact on stock market returns, indicating that investor sentiment fluctuation is one of the factors affecting stock market price trends.

### Contributions

This study offers several contributions to financial researchers. Although previous researchers have explored the relationships between investor sentiment and its various predictors on the basis of a cross-sectional design (Zhang et al., 2017; Ren et al., 2018), this study investigates the two emotional proxy indicators: investor activity (ACT) and stock market leverage level (LEVERAGE). Unlike previous studies that measured investor sentiment at a specific point by the movement of capital between markets (Ding et al., 2017; Lan et al., 2020), this study considers China's securities market and finds that the investor sentiment rate has a more marked impact. To some extent, this means that the effectiveness of China's securities market is insufficient, and there are still many other factors that affect the operation of the market. In view of the fact that the influence of investor sentiment in China's securities market is widespread, the steady and effective development of China's securities market requires securities market regulators to improve laws and supervision (Abbasi and Riaz, 2016). Also, it is important to educate investors on investment concepts. Furthermore, the investor sentiment index constructed in this article provides a risk control perspective.

This research also finds that, although the traditional capital asset pricing model has made a great contribution to market pricing, it is still imperfect, with many of its assumptions being questioned by various empirical studies. First of all, in the field of asset pricing, we believe that, to expand finance theory, behavioral finance and traditional finance need to continue to merge, and the asset pricing framework needs to adapt to the different characteristics of different capital markets. As for China's securities market, continuous improvement and maturity are inevitable, but the speed of the process is vitally related to governmental reform, introducing institutional laws, and improving the education of investors.

### Managerial Implications

This study has implications to the academic community and subsequent researchers. First, studying various factors that affect stock returns, predicting and measuring returns of various types of financial products, and exploring the specific relationship between risk and return are important topics of research. Starting from modern portfolio theory, various models have been proposed to study the risks and returns of financial assets (Guironnet et al., 2016; Li et al., 2018). However, with the development of classic financial theories and the maturity of the capital market, we find that the research results of classic financial theories lack explanatory power for many actual conditions of stock price fluctuations in the market. To bridge the gap between theory and practice, this article recommends that, in addition to the development of classic theories, the academic community should strengthen the analysis of the many practical problems in modeling financial markets. This analysis can consider the perspective of emerging behavioral finance theories, and can promote the integration and systems of capital asset theory development. In any market, as long as the investors are people, psychology and investor sentiment will continue to impact the rate of return.

Second, for market participants, a better understanding of the impact of investor sentimental fluctuations can encourage investors to rationally analyze the market. At present, China's securities market is dominated by private investors without professional, capital and information advantages. Such investors are more likely to blindly speculate on short-term up-and-down trends. Compared with professional investment institutions, their market analysis ability and risk tolerance are low. It is better to encourage investors to make medium- and long-term value investments to reduce irrational behaviors in the investment process (Antony, 2020; Zhang, 2020). Therefore, in the securities market, adding institutional investors would help to stabilize the market structure. Improving the overall investment quality of market traders can reduce the number of noise traders (Peress and Schmidt, 2020) and bring stock prices closer to their actual values.

Third, from the supervising agency perspective, these agencies also use various methods to guide investment participants to establish a good investment philosophy and enhancing investors' emotional awareness and financial knowledge. Whether an institutional investor or an individual investor, all investors should always distinguish between different industries and companies, and between short, medium and long-term investments, so as to treat each differently and accordingly. Investors should focus on the intrinsic value of investment stocks, should be vigilant about risks, and should not one-sidedly pursue high returns while ignoring risks. Individual investors, especially, should treat the market rationally, strive to overcome human weaknesses, and use more comprehensive risk management tools to control risks and their own positions.

### Limitation and Future Research Recommendations

This work has a number of limitations which suggest opportunities for future research. First, because the research budget and time were constrained, this study used only four emotional agent indicators to test the research model. Since the Chinese securities market is an immature emerging market, future studies should collect more data to obtain adequate information about what differences exist between the behavioral financial theory of emerging economies and the traditional financial theory of Western established markets. Second, this article only considers the impact of changes in investor sentiment on stock market returns. In the actual operation of a stock market, changes in stock market returns may in turn affect investor sentiment. This impact should be two-way and should be explored further in the future. Third, this article does not consider the macroeconomic cycle variables to predict and measure the yield of various types of financial products. Nor does it explore the specific relationship between risk and return. Fourth, investor sentiment changes over time according to environmental conditions, making it difficult to determine which specific stocks attract speculators or arbitrageurs. Fifth, this research did not discuss the rationality and ability of stock investors to choose stocks. Since some scholars have proposed that investors have the ability to choose stocks (Li et al., 2016), subsequent researchers still need to further distinguish between smart investors and retail investors.

From the empirical results of this article, we find that foreign capital plays an important role in both the spot market and the futures market in China. Therefore, we recommend that governing authorities should hasten opening up to foreign investment, in order encourage the domestic financial market to align with international standards. This paper can also be used as a reference for the opening up of financial policies in emerging markets. In addition, the empirical results, which have explored the operation methods of foreign investment in periods of high and low sentiment for the futures and spot markets, can be used as a reference for domestic institutional legal entities and general investors. In addition, changes in investor sentiment are a systemic factor affecting stock returns. Investor sentiment thus has a strong ability to predict the volatility of future stock returns. This article also found that investor sentiment in China's Shanghai Stock Exchange (SHCOMP) stock market will significantly affect stock price volatility, with the impact of investor sentiment on volatility being principally through the channel of rewards.

Here are the suggestions for follow-up studies. (1) This study has used the foreign-invested sentiment proxy variables to screen out four indicators. We suggest that technical analysis indicators can be added into these sentiment indicators to see whether it could be more explanatory. (2) Concerning the impact of foreign sentiment indicators on the excess returns and volatility of futures and spot goods, this article only studies the overall market. We suggest that future research efforts should further divide the market into long and short terms, so that the impact of the sentiment indicator on the long market and the short markets can be analyzed separately, while observing the level of impact when the sentiment is extremely high or extremely low. (3) The constructed sentiment indicator can be further used as a predictive model to test whether it has predictive ability for the excess returns in the futures and spot markets.

## Data Availability Statement

The raw data supporting the conclusions of this article will be made available by the authors, without undue reservation.

## Author Contributions

All authors listed have made a substantial, direct and intellectual contribution to the work, and approved it for publication.

## Conflict of Interest

The authors declare that the research was conducted in the absence of any commercial or financial relationships that could be construed as a potential conflict of interest.

## Publisher's Note

All claims expressed in this article are solely those of the authors and do not necessarily represent those of their affiliated organizations, or those of the publisher, the editors and the reviewers. Any product that may be evaluated in this article, or claim that may be made by its manufacturer, is not guaranteed or endorsed by the publisher.
